# Dominant hand, non-dominant hand, or both? The effect of pre-training in hand-eye coordination upon the learning curve of laparoscopic intra-corporeal knot tying

**DOI:** 10.1186/s10397-017-1015-3

**Published:** 2017-07-07

**Authors:** Carlos Roger Molinas, Maria Mercedes Binda, Rudi Campo

**Affiliations:** 1 Neolife – Medicina y Cirugia Reproductiva, Avenida Brasilia 760, 1434 Asuncion, Paraguay; 2 European Academy of Gynaecological Surgery, Leuven, Belgium

**Keywords:** Laparoscopy, Training, Intra-corporeal knot tying, Hand-eye coordination, Learning curve

## Abstract

**Background:**

Training of basic laparoscopic psychomotor skills improves both acquisition and retention of more advanced laparoscopic tasks, such as laparoscopic intra-corporeal knot tying (LICK). This randomized controlled trial (RCT) was performed to evaluate the effect of different pre-training programs in hand-eye coordination (HEC) upon the learning curve of LICK.

**Results:**

The study was performed in a private center in Asunción, Paraguay, by 60 residents/specialists in gynaecology with no experience in laparoscopic surgery. Participants were allocated in three groups. In phase _1_, a baseline test was performed (*T*_1_, three repetitions). In phase 2, participants underwent different training programs for HEC (60 repetitions): G1 with both the dominant hand (DH) and the non-dominant hand (NDH), G2 with the DH only, G3 none. In phase 3, a post HEC/pre LICK training test was performed (*T*_2_, three repetitions). In phase 4, participants underwent a standardized training program for LICK (60 repetitions). In phase 5, a final test was performed (*T*_3_, three repetitions). The score was based on the time taken for task completion system. The scores were plotted and non-linear regression models were used to fit the learning curves to one- and two-phase exponential decay models for each participant (individual curves) and for each group (group curves). For both HEC and LICK, the group learning curves fitted better to the two-phase exponential decay model. For HEC with the DH, G1 and G2 started from a similar point, but G1 reached a lower plateau at a higher speed. In G1, the DH curve started from a lower point than the NDH curve, but both curves reached a similar plateau at comparable speeds. For LICK, all groups started from a similar point, but immediately after HEC training and before LICK training, G1 scored better than the others. All groups reached a similar plateau but with a different decay, G1 reaching this plateau faster than the others groups.

**Conclusions:**

This study demonstrates that pre-training in HEC with both the DH and the NDH shortens the LICK learning curve.

## Background

Today it is generally accepted that the traditional apprentice-tutor model is no longer valid for training all skills necessary for laparoscopic surgery [1]. This agreement is based upon the recognition that, in contrast with open surgery, laparoscopic surgery demands surgical skills and psychomotor skills that not necessarily should be trained together. Indeed, increasing evidences strongly suggests that psychomotor skills must be trained earlier and outside the operating room, and several models have been proposed for this aim [2–7].

Among these validated training models is the Laparoscopic Skills Training and Testing (LASTT) model, developed by The European Academy of Gynaecological Surgery, suitable for training basic laparoscopic psychomotor skills, such as laparoscopic camera navigation (LCN), hand-eye coordination (HEC), and bimanual coordination (BMC) [1, 8–13].

Several studies in these models, including the LASTT model, have sufficiently proved that training improves laparoscopic skills [8–10, 14], which also applies specifically to training in box models as recently reported in a meta-analysis [15]. The majority of the studies base this conclusion upon measurements performed at two or very few points (before and after training). The effect of training however can be better appreciated if several points are taken into consideration, allowing tracking the improvement in performance over time, which is defined as a learning curve [16]. Although learning curves have been observed for many health technologies [17], only recently, they have become regularly used and reported for laparoscopic procedures [10, 18–23].

Following the first system (few measurements before and after training), we have demonstrated in a randomized controlled trial (RCT) performed in a population of residents and specialist in OB&GYN that HEC training with both the dominant hand (DH) and non-dominant hand (NDH) facilitates the acquisition [9] and retention [24] of more complex laparoscopic tasks, such as intra-corporeal knot tying (LICK). The present study was performed to evaluate in detail the learning curves of LICK after different pre-training conditions (no HEC training, HEC training with the DH only, and HEC training with both the DH and the NDH) from non-reported data of the same RCT mentioned above [9].

## Methods

### Participants and venue

The study was carried out in the Centro Médico La Costa in Asunción, Paraguay, and included 60 specialists/residents in OB&GYN with experience in open surgery but with no experience in laparoscopic surgery.

### Instruments, materials, and laparoscopic tasks

The tasks were performed in the LASTT model inserted in the Szabo trainer box with standard laparoscopic instruments (Karl Storz, Tuttlingen, Germany).

#### Task 1 (hand-eye coordination)

The ability to grasp and transport six objects to six specific targets with both the DH and NDH, while navigating a camera was evaluated in a validated model, as described previously [9]. Briefly, with forceps held with the hand being evaluated and the camera with the contra-lateral hand, the six different objects were grasped and transported to their targets in a fixed order. The time for each repetition was limited to 600 s. The task finished either when the last object was transported to its target or when the time limit expired. The task executed with the DH (task 1a) was scored separately than the task executed with the NDH (task 1b).

#### Task 2 (laparoscopic intra-corporeal knot tying)

The ability to perform a LICK was evaluated in a validated model, as described previously [9]. A soft pad with two pre-mounted sutures (vicryl 2-0, 20 cm length), 1 cm between entry and exit sites, and tails equally distributed at both sites was fitted in the Szabo trainer box in a horizontal position. The optic was introduced through a midline port and the needle holders through lower and lateral ports. With a camera fixed at a distance that allowed the visualization of the entire operating field and the needle holders held with the DH and NDH, the tip of the thread was grasped and the thread was pulled through the pad, leaving a 2 cm tail on the opposite side. Then, a double counter-clockwise knot was made, followed by a single clockwise knot, and finally, by a single counter-clockwise knot. The time for each repetition was limited to 600 s. The task finished either when the participant considered he/she completed the knot or when the time limit had expired. Then, the tutor performed a quality control, and only the flat and square knots were considered correctly performed.

### Scoring system

The measurements were based on the time taken for task completion system [6, 15, 21, 25]. Thus, if the task was successfully accomplished within the time limit, the score was the time actually used to execute the task, ranging from 1 to 600. However, if the task was not successfully accomplished within the time limit, a penalty score of 1200 was given.

### Experimental design

The data presented in this study were collected but not reported at the time of an already published RCT [9]. Participants were randomly allocated to three different groups (G1, G2, and G3; *n* = 20 per group). Within each group, they worked in fixed pairs throughout the study. Working sessions of 1–2 h were performed 2–3 times a week in order to optimize the results, as reported by other authors [26]. A supervisor was present at the working station in all sessions to ascertain the set up was correctly ensemble and to score the tasks. The study was carried out in five phases.

- *Phase 1.* All participants received full explanation and video demonstrations of the different tasks and then performed a test (*T*_1_) (three repetitions of each task) to evaluate the baseline skills before any training.
- *Phase 2.* Participants performed different training programs for HEC, according to the group they belong to. G1 trained both the DH and the NDH (60 repetitions of each task in alternating order). G2 trained the DH only (60 repetitions). G3 did not train HEC at all.
- *Phase 3.* All participants performed a second test (*T*_2_), in the same manner than at *T*_1_, to evaluate the skills acquired after HEC training but before LICK training.
- *Phase 4.* All participants performed a standard training program for LICK (60 repetitions).
- *Phase 5.* All participants performed a third test (*T*_3_), in the same manner than at *T*_1_ and *T*_2_, to evaluate the post-training skills.

### Statistics and curve fitting

All statistical comparisons were performed using the GraphPad Prism 6 (GraphPad Software, San Diego, California, USA).

Intergroup differences in age were evaluated with one-way ANOVA, whereas differences in gender, DH side and training status with chi-square tests.

The scores registered at all points were plotted to produce the learning curves for each student (individual learning curves) and for each group (group learning curves). Nonlinear regression models were used to fit the data to the one- and two-phase exponential decay models.

The one-phase exponential decay model is expressed as *Y* = (*Y*_0_ − Plateau) * exp (−K*X) + Plateau. The two-phase exponential decay model is expressed as *Y* = Plateau + SpanFast * exp (−KFast*X) + SpanSlow * exp (−KSlow*X), where SpanFast = (*Y*_0_ − Plateau) * PercentFast * .01, and SpanSlow = (*Y*_0_ − Plateau) * (100 − PercentFast) * .01. *Y* is a dependent variable (score), and *X* is an independent variable (number of the repetition). *Y*_0_ is the *Y* value when *X* is zero (the starting point before any training). Plateau is the *Y* value at infinite times, expressed in the same units as *Y* (the theoretical best score that a subject could achieve with infinite practice). *K*, *K*Fast, and *K*Slow are rate constant, expressed in reciprocal of the *X* units and which measures the steepness of the curve (higher values of *K* indicates faster learning). Span is the difference between *Y*_0_ and Plateau, expressed in the same units as *Y* values. PercentFast is the percentage of the Span accounted for by the faster of the two components. For LICK, the *Y*_3_, which represents the *Y* extrapolated value from *X*_3_ (the first point of the curve immediately after HEC training/before LICK training), was also calculated.

The extra sum-of-squares *F* test was used to evaluate curve fitting (one phase vs. two phase) and if one single curve adequately fits for all groups. The curve parameters (continuous variable normally distributed) are presented as means ± SEM, and parametric test were used for statistical comparisons. For HEC, differences in the DH learning curves between G1 and G2 were evaluated with unpaired *t* test (two groups), whereas differences between DH and NDH in G1 were evaluated with paired *t* test (one group with two curves). For LICK, differences in the learning curves between G1, G2, and G3 were evaluated with one-way ANOVA with Tukey’s Multiple Comparison post-test (three groups). A two-tailed *p* value of <.05 was considered statistically significant.

## Results

The demographics were already reported at the time of the first publication of this RCT [9]. The median age of the participants was 29 years (range 26–45 years), and gender was evenly distributed (50% males, 50% females, *n* = 30 each). The number of specialists (*n* = 20, 40%) was less than the number of residents (*n* = 40, 60%). As expected, the number of right-handed participants (*n* = 55, 92%) was greater than left-handed participants (*n* = 5, 8%). The demographics of the three groups are reported in Table 1. No intergroup differences were detected for any of the parameters.

**Table 1:** Participants’ demographics

	Groups		
	G1 (*n* = 20)	G2 (*n* = 20)	G3 (*n* = 20)
Age (median and range in years)	29 (26–45)	29 (26–37)	32 (27–45)
Gender (%)			
▪ Male	12 (60%)	9 (45%)	9 (45%)
▪ Female	8 (40%)	11 (55%)	11 (55%)
Training status (%)			
▪ Residents	13 (65%)	16 (80%)	11 (55%)
▪ Specialists	7 (35%)	4 (20%)	9 (45%)
Dominant hand side			
▪ Right	19 (95%)	17 (85%)	19 (95%)
▪ Left	1 (5%)	3 (15%)	1 (5%)

Reproduced with permission from Molinas et al. [9]

For both HEC and LICK, the scores registered by each group at *T*_1_, *T*_2_, and *T*_3_ were already reported in a previous study [9]. For the aims of the present study, the scores registered by each participant at all 69 repetitions (R0–R68) were plotted to evaluate the individual and the group learning curves.

### HEC learning curves

The learning curves for the DH were evaluated in G1 and G2, whereas the learning curves for the NDH were evaluated in G1 only.

**Fig. 1:**
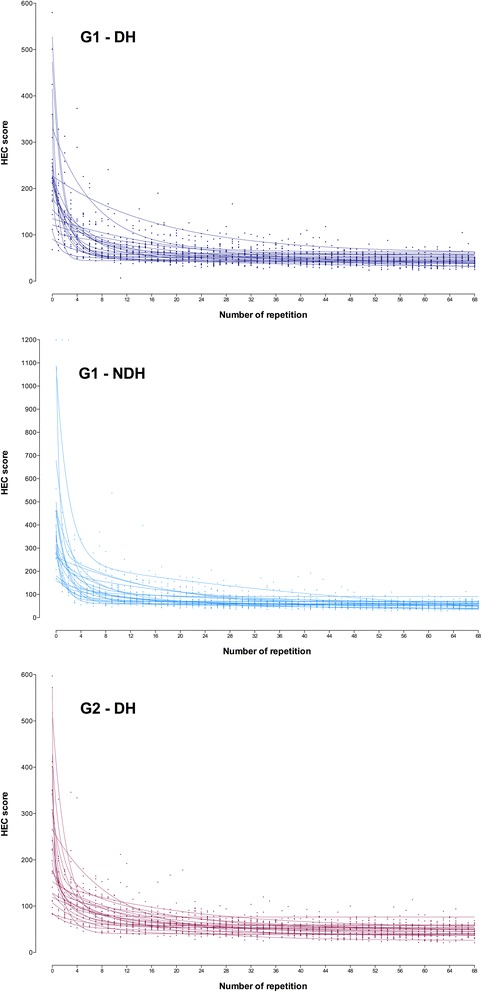
Hand-eye coordination (HEC). Individual learning curves. Participants performed 69 consecutive repetitions (R0–R68) of the task (G1, with both the dominant hand and the non-dominant hand; G2, with the dominant hand only; G3, none). The scores were plotted and individual learning curves were observed, fitting to one- or two-phase exponential decay model according to participants’ performance

Most individual learning curves fitted better the one-phase model, whereas few of them fitted better to two-phase model or were ambiguous (did not fit to any model) (Fig. 1).

**Fig. 2:**
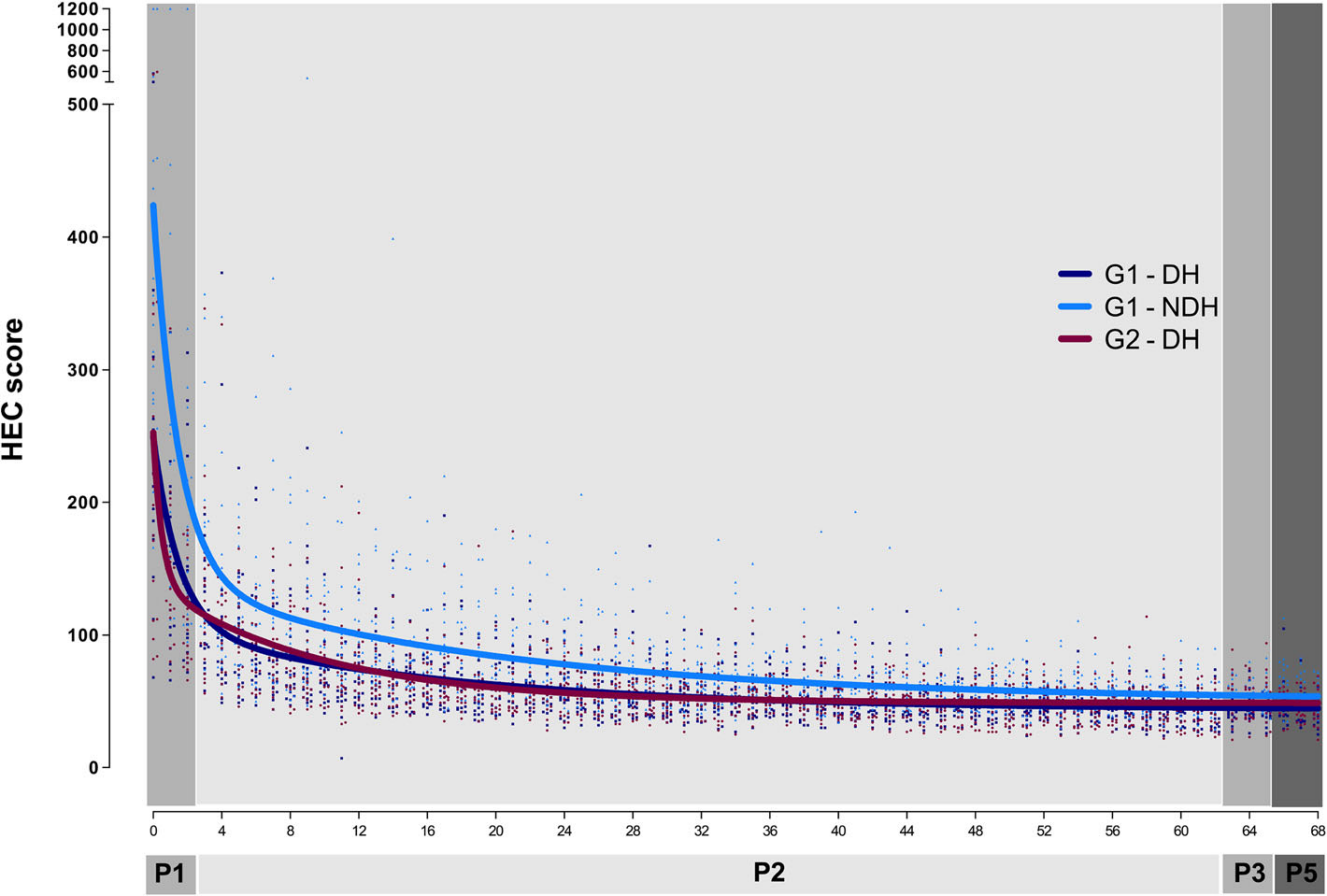
Hand-eye coordination (HEC). Group learning curves. Participants performed 69 consecutive repetitions (R0–R68) of the task (G1, with both the dominant hand and the non-dominant hand; G2 with the dominant hand only; G3, none) during phase 1 (P1): R0–R2, phase 2 (P2): R3–R62, phase 3 (P3): R63–R65, phase 4 (P4): none, and phase 5 (P5): R66–R68. The scores were plotted and group learning curves were calculated. In all groups, the two-phase exponential decay model was the best fitting model

The group learning curves (G1-DH, G1-NDH, G2-DH) fitted better to the two-phase exponential decay model (*p* < .0001 for all comparisons). However, one single type of curve did not adequately fits for G1-DH and G2-DH (*p* < .0001), neither for G1-DH and G1-NDH (*p* < .0001). For the DH, G1 and G2 started from a similar *Y*_0_ (NS), but G1 reached a lower Plateau (*p* = .04), with a higher PercentFast (*p* = .01) and lower *K*Fast (*p* = .02) and KSlow (*p* = .01). In G1, the DH curve started from a lower *Y*_0_ (*p* < .0001) than the NDH curve, but both curves reached a similar Plateau with comparable PercentFast (NS), *K*Fast (NS), and *K*Slow (NS) (Fig. 2 and Table 2).

**Table 2:** Hand-eye coordination (HEC). Parameters of the learning curves

Parameter	Groups		
	G1 - DH	G1 - NDH	G2 - DH
*Y*_0_	250 ± 6*	424 ± 13	253 ± 6
Plateau	44 ± 2^#^	51 ± 6	49 ± 1
PercentFast	70 ± 3^#^	76 ± 3	56 ± 4
KFast	0.67 ± 0.09^#^	0.67 ± 0.09	1.96 ± 0.52
KSlow	0.06 ± 0.01^#^	0.05 ± 0.01	0.10 ± 0.01

Mean ± SEM are presented

G1 trained both the DH and the NDH; G2 trained the DH only; G3 did not train HEC

*p < .05; G1 - DH vs. G1 - NDH

^#^ *p* < .05; G1 - DH vs. G2 - DH

### LICK learning curves

**Fig. 3:**
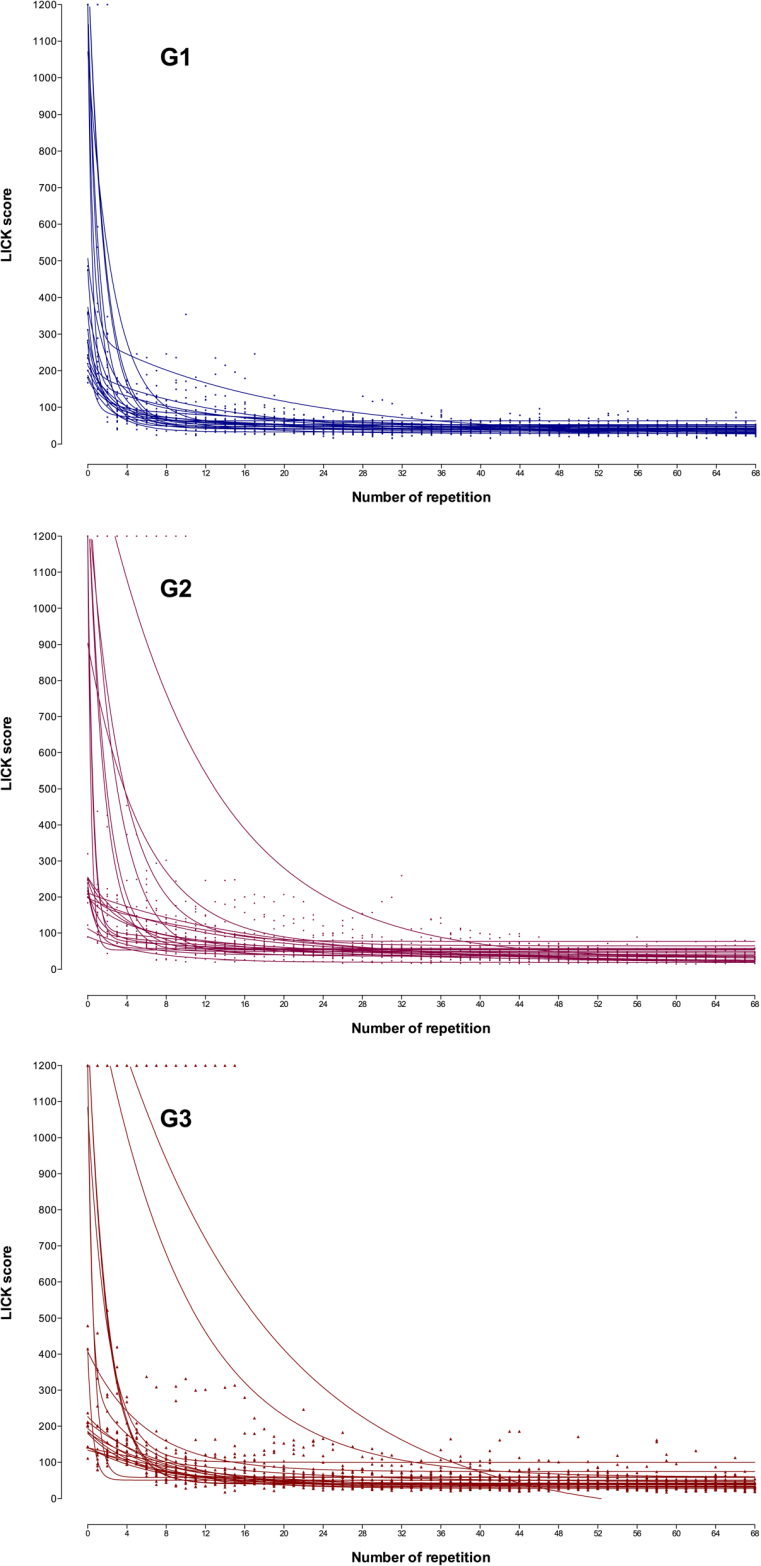
Laparoscopic intra-corporeal knot tying (LICK). Individual learning curves. Participants of G1, G2, and G3 performed 69 consecutive repetitions (R0–R68) of the task. The scores were plotted and individual learning curves were observed, fitting to one- or two-phase exponential decay model according to participants’ performance

Most individual learning curves fitted better the one-phase model, whereas few of them fitted better to two-phase model or were ambiguous (did not fit to any model) (Fig. 3).

**Fig. 4:**
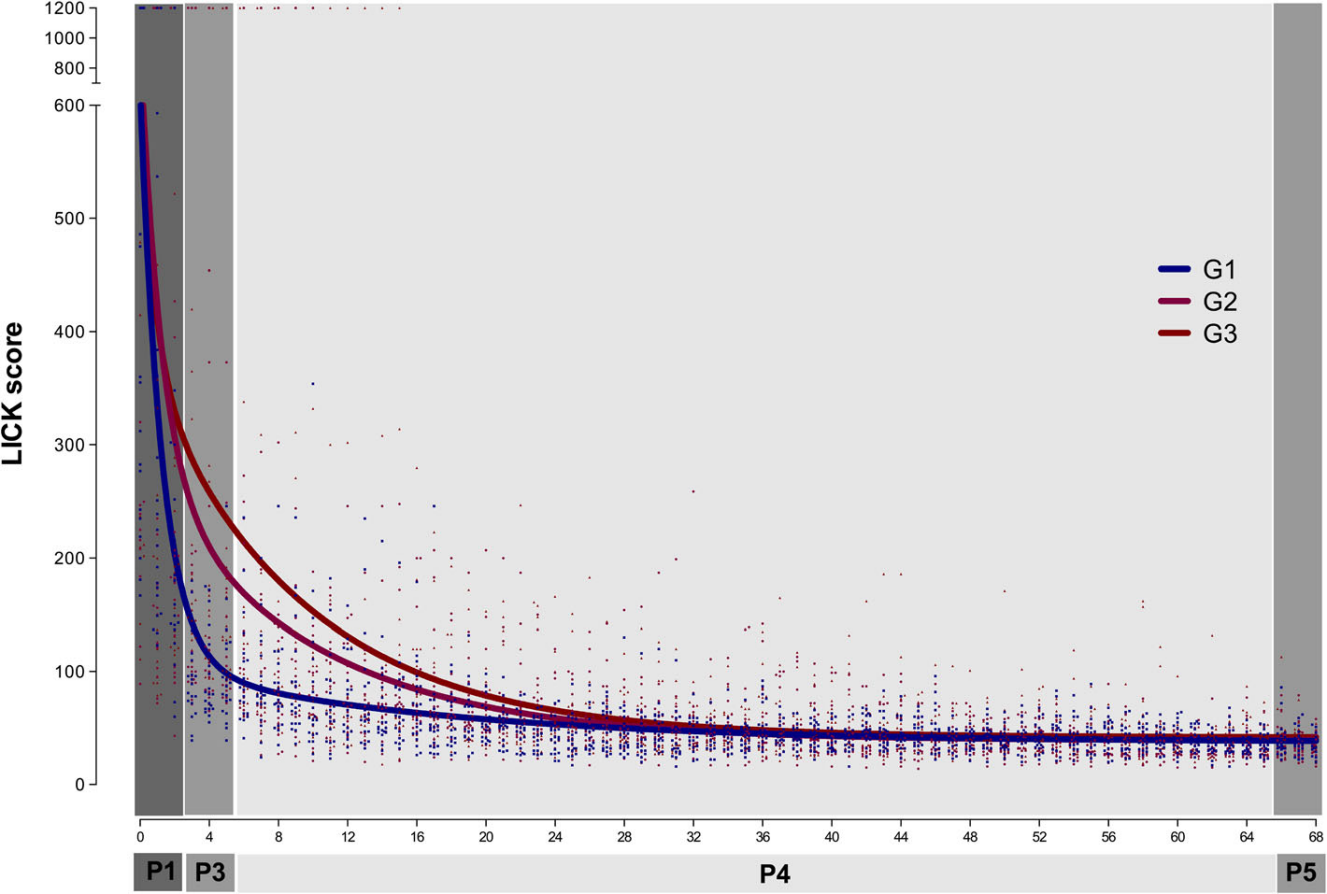
Group laparoscopic intra-corporeal knot tying (LICK) learning curves. Participants of G1, G2, and G3 performed 69 consecutive repetitions (R0–R68) of the task during phase 1 (P1): R0–R2, phase 2 (P2): none, phase 3 (P3): R3–R62, phase 4 (P4): R63–R 65, and phase 5 (P5): R66–R68. The scores were plotted, and group learning curves were calculated. In all groups, the two-phase exponential decay model was the best fitting model

The group learning curves fitted better to a two-phase exponential decay model (*p* < .0001 for all groups) (Fig. 4 and Table 3). However, one single type of curve did not adequately fit for all groups (*p* < .0001). All groups started from a similar *Y*_0_ (NS) and reached a similar Plateau (NS), but the curve decays were different. Indeed, as soon as at *Y*_3_, which represents the extrapolated value from *X*_3_ (the first point of the curve immediately after HEC training/before LICK training), the curve values were already significantly different, G1 scoring lower than G2 (*p* < .05) and G3 (*p* < .05). The PercentFast of G1 was higher than of G2 (*p* < .05) and G3 (*p* < .05), but the differences in KFast and KSlow were not statistically different (Table 3 and Fig. 4).

**Table 3:** Laparoscopic intra-corporeal knot tying (LICK). Parameters of the learning curves

Score	Groups		
	G1	G2	G3
*Y*_0_	615 ± 17	655 ± 29	622 ± 35
*Y*_3_	143 ± 7*	246 ± 13	288 ± 17
Plateau	38 ± 5	39 ± 6	42 ± 6
PercentFast	88 ± 3*	62 ± 9	42 ± 9
KFast	0.79 ± 0.08	0.81 ± 0.22	1.26 ± 0.67
KSlow	0.06 ± 0.03	0.10 ± 0.03	0.11 ± 0.02

Mean ± SEM are presented

G1, G2, and G3 performed the same standard training program for LICK

*p < .05; G1 vs. G2 and G1 vs. G3

## Discussion

This study was performed for insight assessment of the data gathered in the frame of an already published RCT [9] in which changes in the performance of HEC and LICK at three different time points were evaluated. Indeed, in that study, the baseline scores before HEC training (*T*_1_), after HEC training/before LICK training (*T*_2_), and after LICK training (*T*_3_) were evaluated, disregarding the scores registered at each of the 69 points of the study. In this study, the entire dataset was evaluated in order to characterize the learning curves of both HEC and LICK and, more specifically, to determine if pre-training HEC has an influence in the LICK learning curve.

For each task, the real scores of each individual participant were plotted, and obvious individual and group learning curves were observed, which were fitted to the one- and two-phase exponential decay models. An exponential decay equation models many chemical and biological processes. The one-phase model is used whenever the rate at which something happens is proportional to the amount that is left. The two-phase model is used when the outcome measured is the result of the sum of a fast and slow exponential decay, which is also called a double exponential decay. From these curves, the *Y*_0_ (the starting point before any training), the Plateau (the theoretical best score that a subject could achieve with infinite practice), and the Span (the difference between *Y*_0_ and the Plateau) were calculated. From the curves fitted to the one-phase exponential decay model the learning constant (*K*) was also calculated. From the curves fitted to the two-phase exponential decay model the learning constants (*K*Fast and *K*Slow) and the PercentFast (the proportion of the Span accounting for the faster component of the decay) were also calculated.

The individual curves denoted a lot of variability between surgeons, specifically at the beginning of the curves, reflecting the natural heterogeneity in the population (Figs. 1 and 3). The variability, however, decreased significantly at the end of the curves, indicating the positive influence of training regardless the personal characteristics. For both HEC and LICK, some individual curves fitted better to the one-phase exponential decay model, whereas others fitted better to the two-phase exponential decay model regardless the training program.

The learning curves of HEC were characterized for the use of the DH in G1 and G2 and of the NDH in G1. All group curves fitted better to the two-phase exponential decay model. Differences between the DH and the NDH learning curves were evaluated in G1. Although no statistical significant differences were detected in the Plateau, the PercentFast and the learning constants, the NDH curve started from a higher *Y*_0_. This is consistent with our previous report comparing the scores at three specific points, in which the DH scores were better (lower) than the NDH scores before any training (*T*_1_), after HEC training/before LICK training (*T*_2_), and after LICK training (*T*_3_) [9]. We, therefore, hypothesized that the DH curve would decay faster than the NDH curve. Surprisingly, however, the difference detected in this study was observed at the beginning of the curve and not at the Plateau, as would be expected, indicating that appropriate training counteracts the initial differences and that the NDH can achieve skills comparable than the DH. Differences in the DH learning curves of G1 and G2 were also evaluated. Although both curves started from a comparable *Y*_0_, G1 reached a lower Plateau, with a higher PercentFast and lower KFast and KSlow. These better results in G1 can be explained by the fact that the training of the DH and the NDH were performed in alternate and not consecutive order, which could possibly influence positively the learning curve of the DH. Moreover, this can also be explained by some experimental evidences saying that early in training when information about the movement was still spatially encoded and motor programs had not yet been formed, monkeys were able to transfer motor tasks learned with one limb to the opposite limb [27].

The learning curve of LICK was characterized in the control group with no previous training (G3), but because the most important aim of this study was to evaluate the effect of different pre-training conditions, the learning curve was also evaluated in the group that trained HEC with both DH and NDH (G1) and in the group that trained HEC with the DH only (G2). The curves of the three groups fitted better to the two-phase exponential decay model. As expected, all groups had comparable starting points (*Y*_0_). All of them improved their scores at *Y*_3_, which represents the calculated *Y* value at *X*_3_ and which was included to evaluate specifically the impact of the previous HEC training. This improvement was observed in G3, but it was more pronounced in G2 and even more important in G1. In spite of these differences at the beginning of the curve, all groups reached a similar Plateau but again G1 depicted a faster decay, as demonstrated by its significantly higher PercentFast. Since G3 did not train HEC at all, the influence of repetition only cannot be neglected. Since G2 trained HEC with the DH only, the effect of this training is evident. However, the curve characteristics in G1 indicate the relevance of training HEC with both the DH and the NDH in order not only to start the LICK training from a better point but also to achieve proficiency sooner. It is also important to consider an alternative hypothesis: the shorter learning curve observed in G1 was due to the different training volume (G1 performed 120 repetitions in total) and not necessarily due to the training of both hands. In order to define the cause of the positive effect, we would need another group training the DH only for 120 repetitions, but unfortunately, we did not consider such group in the study design.

Our data about HEC learning curves are consistent with previous studies about laparoscopic psychomotor skills in general. Indeed, we have described learning curves after 30 repetitions of HEC with the DH using the same model but with a different scoring system (i.e., number of objects transported in 2 min) in 14 novices and 10 experts [10]. In that study, we reported that experts performed better than novices from the beginning till the end and that after some 20 repetitions, the scores remained similar, but the different parameters of a learning curve were not calculated. Brunner et al. have also described learning curves for 12 basic tasks using a virtual reality model in 12 medical students who performed 30 repetitions of each task in order to define how many repetitions would be necessary to reach the plateau. They reported their data fitted better to a spline model and that a lengthy learning curve existed for novices, which may be seen throughout 30 repetitions and possibly beyond [18].

Our data about LICK learning curves are also consistent with previous studies. Vossen et al. have reported in 29 trainee learning curves with one- or two-phase exponential decay model, the latter fitting their experimental points only marginally better [20]. Zhou et al. [28] and Thiyagarajan et al. [29] have also reported in 20 trainee learning curves with an exponential decay shape. Consistent with our results, the duration of the first knot varied with the previous laparoscopic experience being lower in more experienced trainees [20].

There are few studies evaluating the effect of previous HEC training upon LICK. Consistent with our study, Stefanidis et al. [30] demonstrated in 20 novices that training basic laparoscopic skills (bean drop, running string, block move, checkerboard, and endostitch), all of them representing different tasks for HEC, shortened the learning curve of a more complex laparoscopic task-like suturing. After completing basic skills training, this group achieved proficiency in laparoscopic suturing and knot tying considerably faster and after fewer repetitions (21 ± 8 repetitions) compared with the group with no previous training (50 ± 16 repetitions). They have also claimed the additional benefit of substantial cost savings because the trained group required significantly less active instruction and less overall costs of the suture material. In spite that learning curves were not reported, Fried et al. have also demonstrated in 215 surgeons that training a basic task (i.e., pegboard transfer), which is also a task for HEC, improves significantly the performance of LICK [31].

Although in this study, we did not evaluate effect of surgeon characteristics (age, gender, training status, and DH side) upon the results of the learning curves, in our previous study, we failed to demonstrate any influence of those factors upon the changes in scoring between *T*_1_ and *T*_2_, *T*_2_ and *T*_3_, and *T*_1_ and *T*_3_ [9], which is consistent with other studies showing that the learning curves are not substantially affected by previous exposure to surgery, either by assisting or by watching laparoscopic interventions, nor by personal characteristics, such as leisure activities, eye dysfunction, eye correction, dominant hand, personality, and gender [20, 22, 31]. For gender, however, Thorson et al. have claimed that among medical students, women had a worse performance than men [32], which might be explained by their smaller sample size than in our study (*n* = 32 vs. 60 participants).

It can be argued that one limitation of our study was the scoring system, which was based upon the widely used time taken for task completion system [6, 15, 21, 25]. We have to admit that time alone is not necessarily an accurate assessment of surgical skills and that accuracy and precision should be incorporated into the scoring system. In our system, however, these factors were implicitly incorporated because only objectives correctly achieved were scored. In relation to the basic tasks, this was obvious for both participant and tutor. In relation to LICK, however, knot’s quality could be debatable and differences in participant and tutor validation should be considered. For the aim of this study, only tutor evaluation was considered valid. Unfortunately, we did not correlate both measurements to determine whether students’ assessments improve over time.

On the other hand, we believe that the strength of this study is the measurement of each individual point during the entire training process, which have allowed us to evaluate the learning curves of both basic and advanced laparoscopic tasks. The characteristics of skills acquisition, reported in this study, and of skills retention, reported earlier [24] is consistent with other motor skills acquisition and retention characteristics. Indeed, compelling behavioral and neuro-imaging data suggest that the retention and perfection of skills reflects long-lasting experience-driven changes in the brain’s organization (neural plasticity) [33]. Moreover, extensive motor skill training induces reorganization of movement representations and synaptogenesis within adult motor cortex [34]. Behavioral, functional imaging, electrophysiological, and cellular/molecular studies provide evidence that motor skill learning is a staged process [35]. From the neurological point of view, different mechanisms appear to be active at different times. During training, there is sequential demand for different circuitry. The acquisition phase is characterized by fast (within session) and slow learning (between sessions). Consolidation (i.e., stabilization of novel motor memory) occurs both during and after training. Task complexity may be an important determinant of how “staged” or segregated the process is. Complex motor tasks require several training sessions interspersed with periods of rest and sleep. For these tasks, acquisition and consolidation processes are interlocked, forming a complex sequence of events.

## Conclusions

In conclusion, our study confirms that training improves both basic and advanced laparoscopic skills and demonstrates that the improvement (the decay of the curve) is different according to the individual characteristics, the task complexity, and the training program. This indicates that pre-training of HEC facilitates the acquisition of LICK skills and, moreover, that pre-training of HEC with both the DH and the NDH shortens the LICK learning curve. It remains to be elucidated the potential effect of continues tutoring during training, as suggested by some authors [36], and, moreover, the impact of all these factors upon real surgery in humans.
